# Light People: Prof. Zhanshan Wang

**DOI:** 10.1038/s41377-026-02387-2

**Published:** 2026-07-10

**Authors:** Qiushi Huang, Rongjun Zhang

**Affiliations:** 1 Institute of Precision Optical Engineering (IPOE), School of Physics Science and Engineering, Tongji University, Shanghai, 200092 China; 2 Light Regional Office in Shanghai, Fudan University, Shanghai, 200433 China

**Keywords:** Optics and photonics, Optical physics

## Abstract

At the frontiers of X-ray and high-power laser optics, Professor Zhanshan Wang has made outstanding contributions from fundamental mechanism to fabrication technologies and high performance applications over the last 25 years. As a Professor at Tongji University, he leads the Innovative Research Group of the National Natural Science Foundation of China, pioneered a novel theoretical framework for the synergistic tailoring of spectral response, electric field distribution, irradiation damage and optical loss in thin films optics. He developed high-precision characterization methods for resolving atomic-scale defects in coatings, invented a full-process and quantitative fabrication technology for thin film optics. By establishing premier research platforms and cultivating a highly skilled scientific team, his sustained efforts have greatly improved the performance of X-ray and optical thin-film devices which have been widely applied in synchrotron radiation, high power laser facilities, and space telescope. In this interview, he reflects on the scientific concepts guiding his research on X-ray and laser optics, the philosophy behind cultivating a world-class research team, and his vision for the future of optical science and technology.

**Figure:**
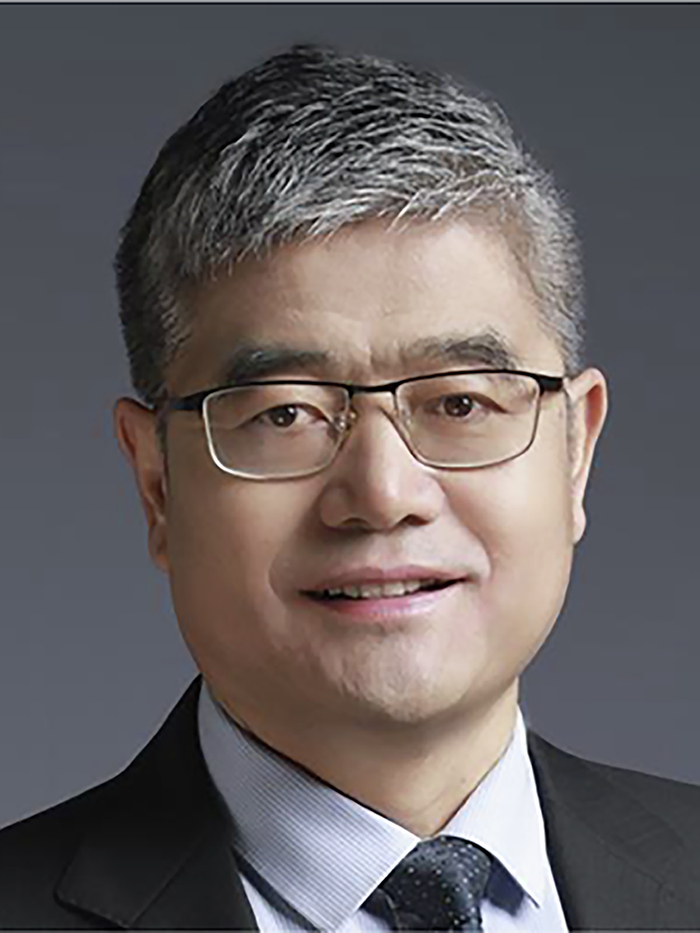

**Zhanshan Wang**, is professor in School of Physics Science and Engineering, Tongji University, China. He has received the National Science Fund for Distinguished Young Scholars and the Changjiang Scholars Distinguished Professor award. He is a Fellow of SPIE (International Society for Optics and Photonics) and a Fellow of the Chinese Optical Society (COS). He serves as the Principal Investigator of the Innovative Research Group supported by the National Natural Science Foundation of China (NSFC) and is the Director of the Key Laboratory of Advanced Micro-Structured Materials, Ministry of Education. Previously, he served as the Dean of the Institute of Advanced Technology and the Chair of the Department of Physics at Tongji University.

He graduated from Nankai University with a Bachelor’s degree in Physics in 1985, received his Master’s degree from the Changchun Institute of Optics, Fine Mechanics and Physics (CIOMP), Chinese Academy of Sciences in 1988. He obtained his Ph.D. in Science from the Shanghai Institute of Optics and Fine Mechanics (SIOM), CAS, in 1996. He joined Tongji University in 2001, where he has been working ever since.

He is an internationally renowned scientist who has long been dedicated to research on X-ray and high-power laser devices and systems. He proposed a novel theory for the performance regulation of optical thin-film devices containing microstructural defects and invented a quantitative fabrication technology for high-performance optical thin-film devices. His technologies for high-power laser and X-ray thin films have reached world-class level, providing a series of key thin-film optics for China’s major scientific engineering projects and high-end civilian instruments. He has led major national projects, including those funded by the NSFC Major Program and the National Key R&D Program of China. He has published over 400 high-level academic papers and holds more than 100 authorized patents. He received two Second Prizes of the National Technology Invention Award (ranked as the 1st and 2nd contributor), the China Patent Gold Award (as the 1st contributor), the First Prize of the Shanghai Technology Invention Award. Many of his graduate students have been selected for prestigious programs such as the National Science Fund for Distinguished Young Scholars, the Excellent Young Scientists Fund, and the Ten Thousand Talent Program. He was selected as one of the inaugural “Shanghai Outstanding Talents” in Shanghai.

## Part A, Core scientific research: stories of technological breakthroughs and innovations

1. X-ray and laser thin film optics: Solving the “bottleneck” problem
  1. X-ray optics: from atomic-scale multilayers to space telescope

**Q1: The high-reflectivity large-size X-ray optical thin film devices developed by your team have broken through the key technology of atomic-level interface defect suppression. You describe this process to “microscopic wall building” (about 600-1200 layers of nanometer-thick films, each layer only 1** **nm thick). Could you specifically share the most difficult technical bottlenecks encountered during the research and development process? And how did you ultimately find solutions such as “atomic fixation”?**

A1: The most challenging work in ultrathin X-ray thin-film research is how to observe and control interfacial defects at the atomic scale. For 1-nm thickness layer, the interfacial defect width is only a few atomic layer; even transmission electron microscopy often cannot unambiguously resolve interfacial interdiffusion and compound formation, not to mention it is destructive. We found that X-ray metrology performed near the working wavelength can provide the highest precision. We developed X-ray standing-wave (XSW) excited fluorescence spectroscopy, and multi-wavelength resonant reflectivity reconstruction methods, enabling accurate, non-destructive characterization of both single and multiple interfaces in X-ray thin films. The depth resolution of the standing-wave method can reach ~0.1 nm, while simultaneously providing information on elemental composition and bonding states. These methods allowed us to identify distinct mechanisms of interfacial intermixing, including compound-reaction-driven mixing and grain-boundary-enhanced diffusion. Guided by these mechanistic insights, we developed targeted strategies for defect suppression of different multilayers. For example, in Pd/Y multilayers, the chemical reaction between the two materials is particularly severe. To address this, we developed a reactive sputtering method to achieve “atomic fixation” by introducing a small amount of nitrogen gas into the inert sputtering gas. The trace nitrides generated in this process passivate the atomic interfaces, thereby suppressing the chemical reactions and intermixing between Pd and Y atoms in the multilayers. These precise control methods were all discovered based on a profound understanding of the underlying mechanisms.

**Q2:** **This achievement won the first prize of the 2024 Shanghai Technology Invention Award and has been applied to national large-scale scientific facilities such as Shanghai Synchrotron Radiation Facility and solar observation in space. The reflectivity of part of the domestically produced devices has reached the international leading level. What practical significance does this breakthrough have for the localization of high-end optical metrology equipment in China?**

**Fig. 1:**
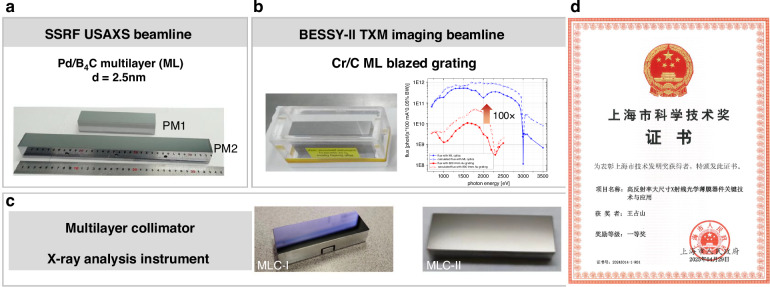
Images of the developed X-ray multilayer optics. **a** multilayer monochromators fabricated and applied inShanghai synchrotron radiation facility (SSRF), **b** multilayer blazed grating joint-developed for BESSY-II beamline and itssignificant enhancement of the photon flux, **c** multilayer collimators fabricated for X-ray analysis instruments, **d** certificate of the 1st prize of 2024 Shanghai Technology Invention Award

A2: Our X-ray thin-film optics are widely applied in large-scale scientific facilities—such as synchrotron radiation and free-electron laser sources, space telescopes, and controllable fusion facilities, as well as the integrated circuit industry and laboratory-scale X-ray analytical instruments. I believe it has two aspects of significance for the development of domestic high-end instrumentation:

First, it breaks technological barriers. For instance, the high reflectance multilayer mirrors developed for the Shanghai Synchrotron Radiation Facility (SSRF) beamline project have been successfully applied in multiple beamline monochromators (Fig. 1). Utilizing these optics, the photon flux at the beamlines was increased by nearly two orders of magnitude compared to traditional crystal monochromators. This advancement enabled the first millisecond-level time-resolved ultra-small angle X-ray scattering characterization in China. This kind of multilayer optics operating under liquid nitrogen cooling were previously supplied by only one company worldwide. Achieving independent R&D means allowing us to truly master these high-end technologies.

Second, it reduces industrial costs and accelerates the R&D process for domestic equipment (Fig. 1). The thin-film monochromators and collimating optics we developed have been applied in domestic X-ray fluorescence spectrometers and diffractometers. Not only are the optics prices significantly reduced, but the delivery time has also been shortened from e.g. one year to six months. This both lowers the R&D costs and final product prices of domestic equipment, and speeds up their time-to-market and technological iteration. It provides critical support and core capabilities for the development of high-end optical metrology equipment.

**Q3: The 46.5-nanometer extreme ultraviolet solar imaging system developed by your team for the SATech-01 satellite has captured the first complete solar image in this wavelength band over the last half century, and it has been included in the International Virtual Solar Observatory. In the research and development of space-grade optical components, how to balance the dual requirements of “high precision” and “resistance to space environmental interference”? What was the feeling when the on-orbit observation was successful?**

A3: The 46.5 nm extreme ultraviolet solar imager (SUTRI, Solar Upper Transition Region Imager) is the first space telescope jointly developed by our institute, and we were the optomechanical system lead unit. The core component of this payload is a reflective imaging system based on multilayers. As space-grade thin-film optical components, they must maintain the same structural precision as ground-based components, with film thickness errors controlled below 0.1 nm. Simultaneously, they must also withstand challenges during launch and orbital operations, including vibration, shock, space radiation, contamination, and other issues.

For instance, some multilayer materials have poor mechanical properties and are prone to fracture under vibration and shock. While controlling coating precision, we also optimized their mechanical properties such as film stress and Young’s modulus to ensure the optics could endure the intense vibrations during rocket launch. Space particle radiation presents another challenge for thin-film optics. We conducted systematic research on the impact mechanisms of protons, electrons, and atomic oxygen with different energy on film structure and performance, ultimately selecting materials with optimal optical and radiation resistance properties for component fabrication. It is only through the dual technological guarantees of “high precision” and “resistance to space environment” that we can confidently claim the payload’s flawless launch and operation.

**Fig. 2:**
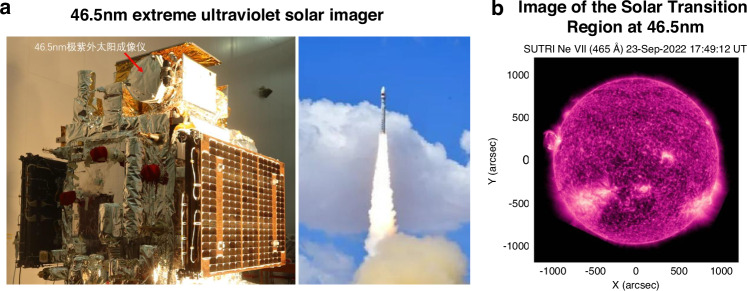
The 46.5 nm extreme ultraviolet solar imager and its observation result. **a** picture of the solar imager system (indicated by arrow) after integrated on the satallite platform, **b** image of the solar transition region observed at 46.5 nm wavelength

SUTRI payload was successfully launched on July 27, 2022, when we received the first 46.5 nm solar image in early September, the feeling was indescribable—we knew every effort was worthwhile (Fig. 2).

(2) Laser thin films and precision imaging: tackling technical challenges across multiple fields

**Q1:** **You have proposed a new concept of “full-process quantification” for laser thin film production, establishing a complete platform from substrate manufacturing to post-processing. The related technology has won the second prize of the State Technological Innovation Award and the China Patent Gold Award. What is the core innovation of this concept? How do you solve the common problem of “high damage threshold” for laser thin films?**

A1: Building upon my research foundation in X-ray thin films, and driven by national needs, I began studying laser coatings. I believe the core of the “full-process quantitative” laser coating fabrication philosophy is a profound understanding of the scientific challenge of laser-induced damage. This perspective, I think, is shaped by my dual research experiences in both academic and institutional settings—requiring a dual comprehension of both the engineering requirements and the underlying physical mechanisms of a problem. This suits for both X-ray coatings and laser coatings.

**Fig. 3:**
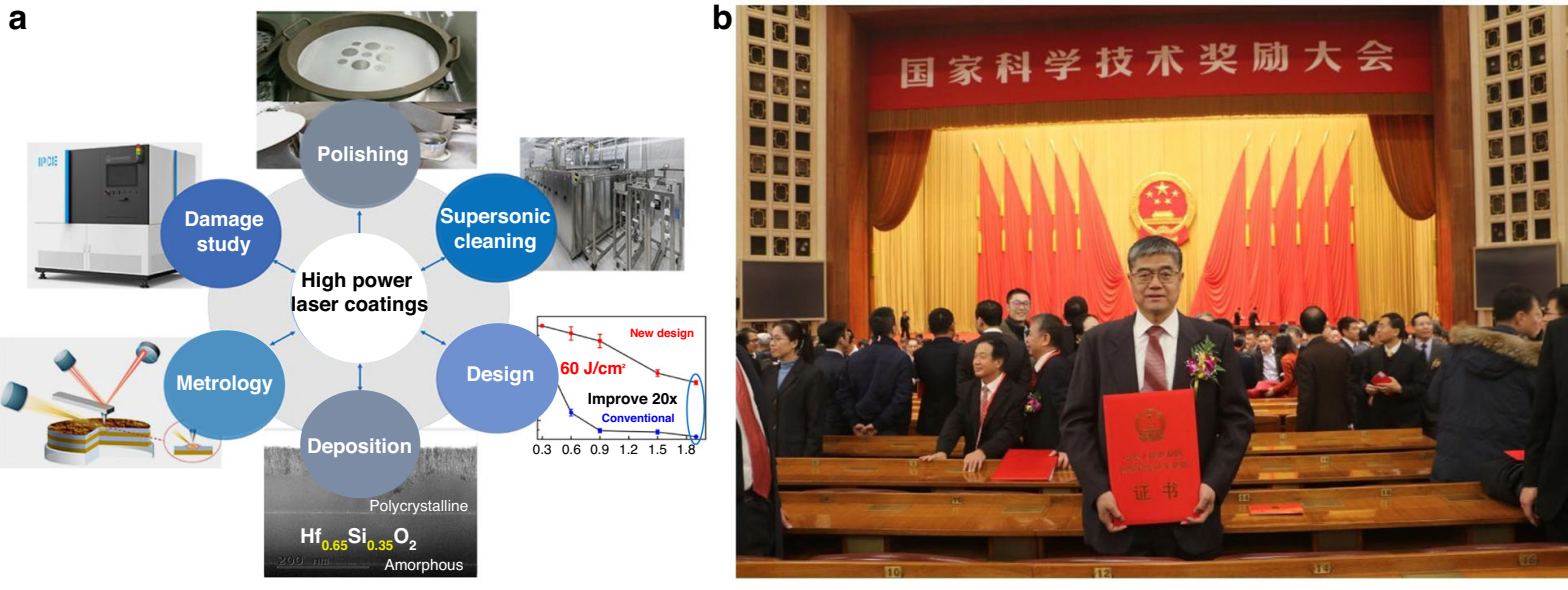
Full-process quantitative development of high-power laser thin film optics. **a** the full-process control chart including from design, cleaning, polishing, to deposition, metrology and damage study, **b** photo of Professor Zhanshan Wang receiving the second prize of the National Technology Invention Award in 2019

Guided by this approach, to master the quantitative laws governing laser coating damage, we proposed using controllable artificial defects to simulate real-world scenarios for quantitative damage studies. This enabled us to clarify single-factor patterns affecting damage thresholds and revealed that electric field enhancement is the primary factor influencing damage thresholds. Informed by these findings, we developed full-process quantitative defect control technologies. These include coating design methods to suppress localized electric field enhancement, novel nanocomposite materials to eliminate electric field hotspots, and a series of substrate polishing and etching techniques (Fig. 3). Through comprehensive control and optimization across design, materials, and every process step, we solved the critical challenge of achieving high damage thresholds.

**Q2:** **The team’s four-channel and eight-channel soft X-ray KB microscopic imaging systems have been successfully applied, and significant progress has been made in the field of extreme ultraviolet and soft X-ray polarization measurement. What specific roles have these technologies played in basic scientific research and national major projects? Are there any application cases that impress you?**

A2: Extreme ultraviolet (EUV) and soft X-ray polarimetry form the foundation for various synchrotron techniques including magnetic circular dichroism, magnetic imaging, and spin-resolved spectroscopy—all requiring high-efficiency polarization optics. Conventional periodic multilayers only enable narrowband polarization control, limiting practical applications. To overcome this, we pioneered broadband polarization devices using non-periodic multilayer structures, achieving the first broadband full-polarization detection in synchrotron beamlines. This technology has been applied at Beijing Synchrotron Radiation Facility, BESSY-II in Germany, and Diamond Light Source in UK. Our broadband polarization analysis method using non-periodic multilayers has been incorporated into the third edition of the Handbook of Optics published by the Optical Society of America.

X-ray multichannel microscope represents our transition from core thin-film components to integrated optical systems, developed for diagnosing strong-field physics. This requires simultaneous acquisition of spatial, temporal, and spectral information of the plasma. We thus proposed a novel X-ray spatiotemporal-spectral multidimensional imaging technique by integrating thin-film dispersive elements with multichannel Kirkpatrick-Baez (K-B) mirrors. To address the challenge of integration of soft X-ray systems under atmospheric condition, we developed a precision alignment method using dual energy (hard/soft X-ray) multilayer optics. This breakthrough enables rapid and accurate deployment of X-ray multichannel imaging systems from laboratory to complex operational environments.

**Q3:** **The precision of modern optical manufacturing is becoming increasingly high. For example, the X-ray multilayer mirror you produced for the Shanghai Synchrotron Radiation Facility has an equivalent film thickness uniformity error of tens of picometers. You have also applied artificial intelligence technology to film quality measurement and control. Is the integration of AI and traditional optical manufacturing an important direction for future technological advancement? What integration challenges are currently being faced?**

A3: As optical manufacturing precision advances to sub-nanometer/atomic levels, small perturbations during coating and polishing processes can lead to significant error accumulation. Traditional physical models struggle to accurately describe such complex nonlinear effects, whereas artificial intelligence (AI) holds substantial potential here. Currently, many studies including our own, are exploring AI applications in thin-film design and the inverse retrieval of structural-performance metrics. I believe this field remains in its infancy, facing multiple challenges: In which specific scenarios can AI methods outperform traditional methods? How can learning algorithm and physical model be better integrated? How to use limited experimental data be collected to train AI models? These questions demand further explorations.

## Part B, Academic career: team building and field contribution

**Q1:** **You have presided over multiple national-level projects such as the National Science and Technology Major Special Projects, 973 Program projects, and key research and development plans, and you have also participated in the preparation of multiple national standards for optical thin films. In the process of promoting the standardized and normalized development of disciplines, what do you think is the “weak link” that needs to be addressed most urgently in China’s optical field?**

A1: Standardization and normalization in precision optics constitute a critical link in transitioning from ‘laboratory validation’ to ‘engineering-scale mass production.’ This is indispensable for integrated circuit manufacturing, large-scale telescope development, etc. I think a significant gap currently exists is ultraprecision optical metrology methods and inspection instruments. We still rely heavily on imported high-end equipment and core technologies in these areas. If the ‘metrology ruler’ is from others, it becomes difficult to secure standard-setting authority. Another critical gap may lie in the absence of comprehensive lifecycle-spanning process specifications and quality control systems. This deficiency further exacerbates the disconnect between fundamental research and large-scale industrial deployment. Resolving this challenge demands our collective effort and collaborative innovation.

**Q2:** **You have conducted collaborative research at King’s College London and the Fraunhofer Institute for Applied Optics in Germany. How has international academic exchange inspired your scientific research ideas? What are the current research hotspots and competitive landscape in the global optics field? How should Chinese scholars enhance their voice on the international stage?**

A2: I think China’s optical capabilities today have progressed dramatically since my time at King’s College London two decades ago. The international landscape and conditions are fundamentally different, and our international standing continues to rise. Yet, our research remains predominantly catch-up oriented, lacking truly original breakthroughs. To improve this, we should first encourage pioneering research—only transformative, field-leading achievements can bring genuine influence. Operationally, this requires moderating short-term quantitative metrics for research evaluation. We should incentivize frontier exploration, allowing failures while demanding rigorous failure analysis. Crucially, cutting-edge research thrives on global engagement: identifying high-value problems and collaborating with world-leading partners. Journals like Light has done great contributions here, providing premier platforms for curating and disseminating optical breakthroughs.

**Fig. 4:**
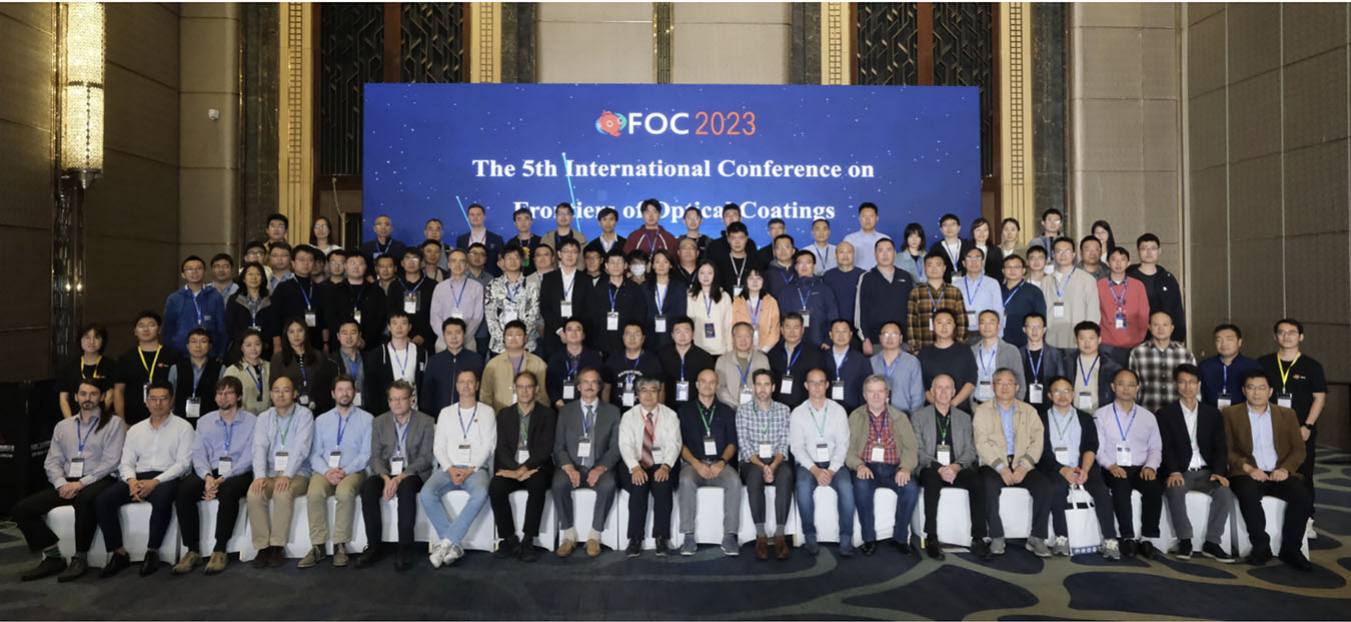
The 5th International Conference on Frontiers of Optical Thin Films, organized by Professor Wang Zhanshan in 2023

Meanwhile, in precision engineering domains—integrated circuits, space optics, and other strategically contested fields—barriers are unavoidable. Yet even here, we must persistently seek collaborative openings and maintaining an open, cooperative mindset. We have consistently hosted Asia’s highest level international conference in optical thin films—The International Conference on Frontiers of Optical Coatings (FOC) (Fig. 4). The Fifth Edition in 2023, to continuously advance global exchanges and collaborations.

## Part C, Academic aspirations and career choices

**Q1:** **You graduated from Nankai University with a bachelor’s degree in Physics, and then pursued your master’s and doctoral degrees at the Changchun Institute of Optics, Fine Mechanics and Physics, Chinese Academy of Sciences, where you delved deeply into the field of optics. How did this experience inspire you to focus on research directions such as X-ray optics and laser thin films in your subsequent career? Why did you firmly choose optics as your lifelong research field at that time?**

A1: I believe university education is incredibly helpful in broadening one’s mind. The wide array of majors—ranging from mathematics, physics, chemistry, and biology to Chinese literature, economics, and management—truly expanded my horizons. During my time at Nankai University, the Institute of Modern Optics was established, and it was there that I first received my training in optics. After completing my undergraduate studies, I wanted to pursue further education closer to home. The Changchun Institute of Optics, Fine Mechanics and Physics (CIOMP) is known as the “cradle of optics” in China, so it naturally became my ideal choice.

My supervisor at CIOMP was Mr. Chen Xingdan. He did pioneering work in the measurement of optical radiation from nuclear explosions in China and later led research on components and systems for short-wave optics, such as X-rays. The scientific attitude and selfless spirit of Mr. Chen’s generation had a profound impact on me and were significant factors in my decision to pursue optics as my career path.

**Q2: From an intern researcher at the Changchun Institute of Optics, Fine Mechanics and Physics to a professor at Tongji University and the dean of the Advanced Technology Research Institute, which stage or event in your decades-long academic career has given you the most sense of achievement? What is the core motivation that sustains your continuous deepening of research in optics?**

**Fig. 5:**
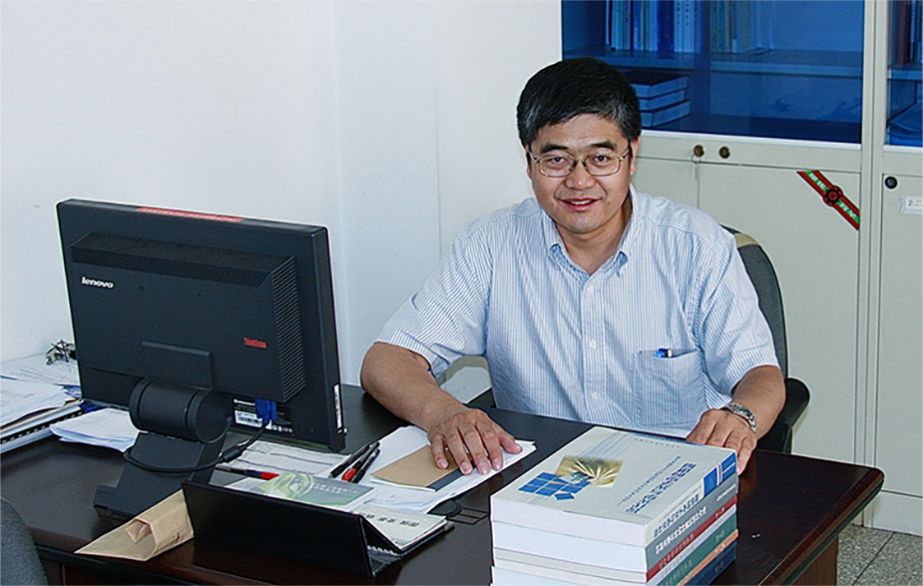
In 2001, Professor Wang Zhanshan joined the Department of Physics at Tongji University

**Fig. 6:**
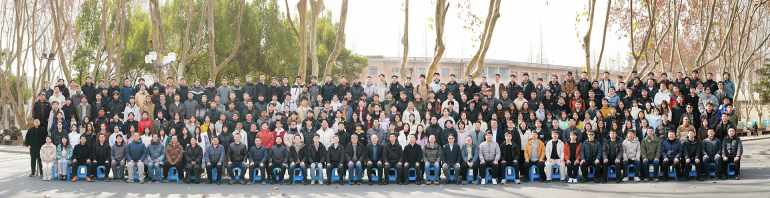
Group photo of Professor Wang Zhanshan’s team in early 2026

A2: I arrived at Tongji University in 2001, and it has now been 25 years. I vividly remember that when I first started, my office and lab were located in the old Telecom Building at the Siping Road Campus. Back then, I was essentially starting from scratch, building up our equipment and facilities from the ground up (Fig. 5). Over the past 25 years, our team has relocated our laboratories four times, and this year, some of our faculty members are preparing for a fifth move. Today, our team has grown to include 45 faculty members and 247 graduate students, with research equipments exceeding 300 million RMB. We have made certain contributions to the state in the fields of X-ray multilayers, high-power laser coatings, and system technologies. I believe that this 25-year journey of development and the achievements we’ve made are my greatest source of fulfillment (Fig. 6).

The driving force that has sustained my team and me through these 25 years of progress is our dedication to the cause of optics—truly loving what we do. I recall a question posed by Tongji’s former president: “What is the difference between excellent and pursuing excellence?” My answer is this: Excellent is an evaluation of what you have already achieved, whereas pursuing excellence is a continuous evolution—a mindset of always moving forward.

## Part D, Outlook and legacy of messages

**Q1: In response to the major strategic needs of the country, what do you think are the core development directions for X-ray optics, laser thin films, and other fields in the next 5-10 years? What technical bottlenecks still need to be overcome?**

A1: In the field of X-ray optics, I think synchrotron radiation and free-electron laser sources with ultra-high brightness and coherence, represent the pinnacle of X-ray light source performance. Given their close ties to frontier applications in materials science, life sciences, and energy, these large-scale scientific facilities will continue to serve as vital drivers and sources of innovation for the development of advanced X-ray optics. Furthermore, applications under extreme conditions—such as high energy density plasma and space observation—will also spur the emergence of new technologies. These will necessitate X-ray optical components with higher precision, more complex configurations, and enhanced radiation resistance to support the development of next-generation observation techniques and applications. The advancement of X-ray optical technologies in high-tech industries like integrated circuits will also constitute a significant direction.

In the field of laser thin films, the pursuit of ultimate performance remains the primary direction. Films with ultra-low absorption and ultra-low scattering are still the goals being constantly sought after in laser equipment and precision measurement. Specifically, the long-term performance stability of deep ultraviolet (DUV) laser films is pivotal for the stable operation of DUV lithography systems, representing a critical demand in high-end integrated circuit equipment. The integration of metasurface and other micro-nano structures with thin films represents a frontier development direction. This approach can significantly enhance the dimensions of light field modulation and transmission capabilities of thin film devices. Consequently, design models for micro-nano thin film devices, cross-scale manufacturing, and computational sensing methods integrating multi-dimensional modulation will be future research hotspots. Furthermore, Artificial Intelligence (AI) will converge with thin film devices, elevating the level of intelligence across the entire chain of laser film development: design, fabrication, and characterization.

**Q2: As an outstanding scholar who graduated from the Changchun Institute of Optics, Fine Mechanics and Physics, Chinese Academy of Sciences and now associated with the Light journal, what are your expectations for the development of this journal? What advice do you have for young scholars and students in the field of optics regarding scientific research or career choices?**

A2: With its impact factor, Light has established itself as a premier international journal in optics. We anticipate it will exert enduring influence as a defining benchmark for global optical frontiers.

To young scholars and students, my personal advice is to set higher goals and cultivate a mindset of pursuing excellence. While real-world constraints and evaluation metrics exist, I think one’s orientation and goal are pivotal to lifelong development—a crucial consideration when making choices. Regarding career decisions, I think that commitment to a research path sometimes outweighs fleeting interests.

**Q3: You have always emphasized that “scientific research should be closely aligned with national needs”. In your decades-long scientific research career, have there been moments when you found it difficult to persist? What beliefs have supported you in overcoming challenges along the way? If you had to summarize your academic philosophy in one sentence, what would it be?**

A3: When we first entered the field of laser coatings, we purchased our first e-beam coater on credit, repaying the company over five years. Back then, my student asked me: “Can we really pull this off? Will we be able to repay the debt?” I remained steadfast in my conviction that we would succeed. First, because our approach was grounded in systems engineering principles; second, I believed that by mastering the scientific fundamentals, we should be able to solve this problem. Obstacles were inevitable—but we were prepared to tunnel through mountains and build bridges across rivers.

For the Institute of Precision Optical Engineering (IPOE) at Tongji University, I crafted a cultural manifesto embodying my academic philosophy in few words:

Loyalty; Excellence; Collaboration; Innovation.

